# The carbon dioxide laser: an alternative surgery technique for the treatment of common cutaneous tumors in dogs

**DOI:** 10.1186/1751-0147-56-1

**Published:** 2014-01-07

**Authors:** Joanna Paczuska, Zdzisław Kiełbowicz, Marcin Nowak, Agnieszka Antończyk, Rafał Ciaputa, Jakub Nicpoń

**Affiliations:** 1 Department of Surgery, Faculty of Veterinary Medicine, Wrocław University of Environmental and Life Science, pl. Grunwaldzki 51, 50-366 Wrocław, Poland; 2 Division of Pathomorphology and Veterinary Forensics, Department of Pathology, Faculty of Veterinary Medicine, Wrocław University of Environmental and Life Science, ul. C.K.Norwida 31, 50-375 Wrocław, Poland

**Keywords:** CO_2_ laser surgery, Ablation, Skin tumors, Dogs

## Abstract

**Background:**

Tumors of the skin and subcutaneous tissue are the largest group of canine neoplasms. Total excision is still the most effective method for treatment of these skin tumors. For its universal properties the carbon dioxide (CO_2_) laser appears to be an excellent surgical instrument in veterinary surgery. Laser techniques are alternatives to traditional methods for the surgical management of tumors. The aim of this study was to compare various types of laser techniques in skin oncologic surgery: excision, ablation and mixed technique and to suggest which technique of CO_2_ laser procedure is the most useful in particular case of tumors in dogs.

**Findings:**

The study was performed on 38 privately-owned dogs with total number of 40 skin tumors of different type removed by various CO_2_ laser operation techniques from 2010–2013. The treatment effect was based on the surgical wound evaluation, the relative time of healing and possible local recurrence of the tumor after 3 months post surgery. Local recurrence was observed in two cases. The study showed that in 30 cases time needed for complete resection of lesions was less than 10 minutes. Time of healing was longer than 12 days in 6 cases (42.8%) with tumor excision and in 14 cases (87.5%) where excision with ablation technique was performed.

**Conclusions:**

The advantages of the CO_2_ laser surgery were better hemostasis, precision of working, non-contact dissection, less instruments at the site of operation and minimum traumatization of the surrounding tissues.

## Findings

Laser light wavelength and frequency determine the way the laser light interacts with its target tissue. The carbon dioxide (CO_2_) laser emits coherent, collimated and monochromatic beam of light at a wavelength of 10600 nm. Wavelengths of laser light are highly absorbed by water followed by hemoglobin, melanin, and some proteins resulting in photothermal laser-tissue interaction [1]. High degree of absorption of CO2 laser light by water allows precise cutting of tissue via vaporization of the intra- and extracellular fluid and destruction of the cell membranes [2]. The CO_2_ laser can seal and coagulate small blood vessels (up to 0.5 mm), lymphatics and nerve endings resulting in better visualization [3-6]. Non contact mode of excision with laser can reduce intraroperative wound contamination by tumor cells [5].

The aim of this study was to compare different laser techniques in oncologic surgery of skin lesions: excision, ablation and mixed technique and to reveal the most useful CO_2_ laser technique for removal of skin tumors in dogs, depending on size, localization and malignancy.

Our study was conducted in the Surgery Department of Veterinary Medicine at the Wrocław University of Environmental and Life Sciences (Poland). Thirty-eight dogs with skin tumors were evaluated with 3-month follow-up after surgical treatment. The resection of the total number of 40 skin tumors of different types was performed (Table 1). The type of laser surgery technique used depended on localization, tumor size and suspected malignancy (Table 2). Excision was carried out in 14 cases, ablation in 10 cases, and mixed technique in 16 cases. All surgeries were performed with Eraser-C CO_2_ laser with articulated arm delivery system. Choice of surgical technique and power settings were based on the surgeon’s previous experience and references from other authors [4,5].

**Table 1:** **Number of tumor cases removed with CO**_**2 **_**laser depending on diameter, origin and malignancy**

**Diameter [cm]**	**<0.5**	**0.5-1.5**	**>1.5**
**Melanoma**	1 (Mm)	1 (Mm)	0
**Histiocytoma**	0	3 (Mb)	3 (Mm)
**Squamous cell carcinoma**	1 (Nm)	1 (Nm)	2 (Nm)
**Adenoma**	2 (Nb)	3 (Nb)	3 (Nb)
**Papilloma**	4 (Nb)	2 (Nb)	0
**Mastocytoma**	0	1 (Mb)	3 (Mm)
**Adenocarcinoma**	0	1 (Nm)	3 (Nm)
**Hemangioma**	0	1 (Mb)	0
**Fibroma**	4 (Mb)	1 (Mb)	0

*M* tumor of mesenchymal origin, *N* tumor of epithelial origin, *b* benign, *m* malign.

**Table 2:** **Number of cases of CO**_**2 **_**laser surgery technique applied for resection of different size of cutaneous tumors**

**Diameter [cm]**	**< 0.5**	**0.5-1.5**	**>1.5**
**Excision**	0	2	12
**Ablation**	10	0	0
**Excision with ablation**	2	12	2

Preanesthetic sedation was obtained with medetomidine 10 mg/kg body weight and butorphanol 0.2 to 0.4 mg/kg injected intramuscularly. For local anesthesia 2% lidocaine was used. When longer anesthesia was required intravenous injection of propofol at a dose of 2–5 mg/kg was administered.

**Figure 1:**
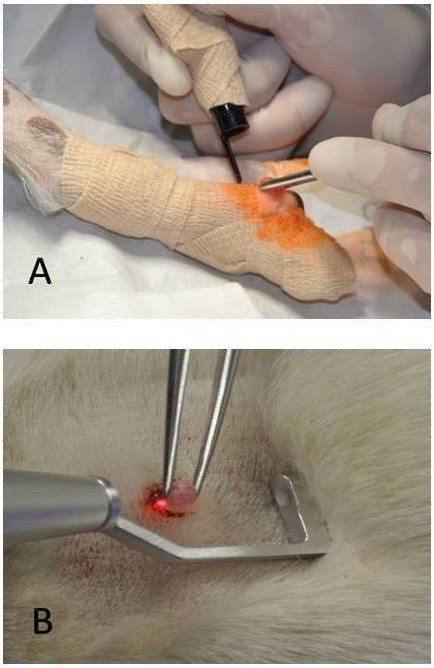
**Tumor excision performed with CO**_**2 **_**laser. A** – CO_2_ laser with distance tip should be located perpendicular to the cut surface, proper tissue tension is important to expose new tissue to laser energy. **B** – CO_2_ laser with backstop tip that protects against uncontrolled beam incidence.

Excision was made in 12 cases of tumors greater than 1.5 cm diameter, and 2 cases of tumors ranging from 0.5 to 1.5 cm in diameter. Prior to the incision, margins were marked using 4 W continuous wave (CW) mode and 0.2 mm spot diameter (SD) guideline around the mass. Excision was performed using 8–12 W power output in CW mode and 0.2 mm SD. The skin margin was incised down to the subcutaneous tissue along the guiding line. During the excision the mass was retracted to ensure adequate tissue tension (Figure 1). To avoid collateral thermal tissue damage any char formation was removed with saline gauze. Vessels of a diameter above 1 mm were ligated with absorbable 3–0 monofilament. To ensure that no untreated tissue had been left, a crosshatched pattern was used to cover areas of the tumor bed, with multiple passes of the CO_2_ laser beam in perpendicular directions with 4 W power output in CW mode and 0.4 mm SD. After excision, the post surgical wound was sutured with 3–0 absorbable monofilament with non-traumatic needle.

Ablation was made in 10 cases of neoplasm measuring less than 0.5 mm in diameter. The target tissue was painted and vaporized using 6–12 W, CW mode and 0.2- 0.4 mm SD. Char was wiped away. Ablation of underlying tissue and a char wipe was repeated until total removal of the masses. The wound was left for second intention healing without suturing.

Mixed technique was administered in areas where total removal by excision was hard to achieve or biopsies for histopathological evaluation were required. Detachment of the tumor mass was maintained by excision with 8–12 W power output in CW mode and 0.2 mm SD. Underlying mass and 0.5 mm margins where ablated with CW mode setting ranging from 6–12 W power output and 0.4 mm SD.

Postsurgical evaluation of the wound was performed immediately after and at post-operative days 3, 6, and 12. The wound was evaluated for the presence of exudate or edema. The area of tumor extirpation was evaluated for local tumor recurrence after three months.

Total time needed for tumor removal in 16 cases was less than 3 minutes, in 14 cases between 3 and 10 minutes, and in 10 cases the surgery lasted more than 10 minutes (Table 3). Time of ablation was less than 3 minutes in all cases. The time needed to remove the tumor was dependent on its size and skills of the surgeon. In our study, the total time of the procedure was less than 10 min regardless of the technique used for 75% of cases.

**Table 3:** Time of procedure in groups with different surgery technique: ablation, excision and ablation with excision

**Time of procedure [min]**	**Excision [%]**	**Ablation [%]**	**Excision/Ablation [%]**
**< 3**	14.9	100.0	25.0
**3-10**	57.1	0	43.7
**>10**	28.0	0	31.3

In the group with tumors removed by surgical CO_2_ laser excision, the healing process in 6 cases (42.8%) was longer than 12 days. Local recurrence has been observed in one patient with squamous cell carcinoma localized on the ear.

After tumor removal by ablation with CO_2_ surgical laser, the healing process was shorter than 12 days. No local recurrence was observed.

The healing process was longer than 12 days in 14 cases (87.5%) after tumor removal by mixed operation technique. Local recurrence was found in one case of mastocytoma localized on the distal part of the hind leg.

**Figure 2:**
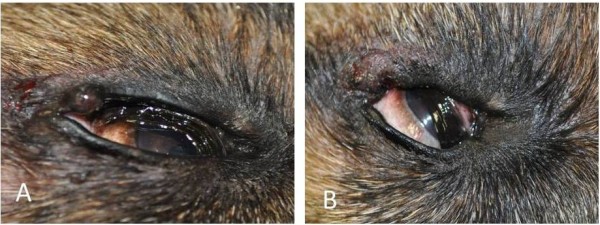
**Meibomian gland tumor localized in the upper eyelid of an 11-yearsold German Shepherd. A** – before CO_2_ laser ablation of the tumor. **B** - few minutes after CO_2_ laser ablation of the tumor.

The ablation technique was chosen only in cases of tumor size less than 0.5 cm, when wide margins of excision weren’t required or possible to maintain, such as masses localized on the eyelid (Figure 2). CO_2_ ablation is useful in all cases of small benign lesions. Ablative techniques should be considered only when complete excision with clean margins is not possible [5].

Excision with CO_2_ laser is a very useful surgery technique in cases of larger tumors when margins of resection and histological evaluation of the excised tissue are required. Excision of all abnormal tissue may be applied as readily as they do to conventional surgery [7]. Improved visibility through better hemostasis provides greater confidence of complete excision and time saving.

**Figure 3:**
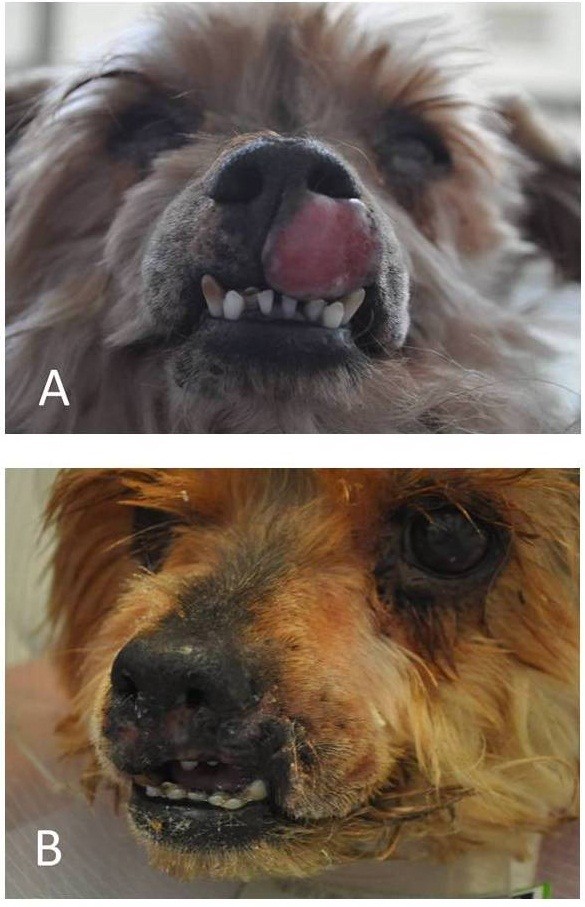
**8-years-old mixed breed dog with histiocytoma localized in the upper lip and nose area. A** – before tumor removal with CO_2_ laser excision-ablation technique. **B** - 6 days after tumor removal with CO_2_ laser excision-ablation technique.

Excision with ablation technique is useful when excision with wide margins is not possible (Figure 3). Excision with ablation was used to remove masses localized on limbs, eyelid, nose, lip and ears, particularly in cases of malignant tumors such as melanoma, squamous cell carcinoma, and mastocytoma with uncertainty about complete excision of all neoplastic cells.

Proper laser surgery technique with adequate power density delivered to the target tissue has a very important influence on thermal damage to adjacent structures and consequently on wound healing [2,7]. An average zone of thermal damage after laser incision in soft tissues is <0.6 mm [2,8,9]. General effects of laser on soft tissues are correlated with the delivered amount of energy or power density, which is determined by laser power outage (watts) divided by the laser tip spot size (cm^2^). A thin layer of char is surrounded by an area of coagulative necrosis which is removed during the normal healing by exudation. Larger zones of reversible edema and inflammation develops within minutes or hours post surgery but later resolves [6]. In our study edema occurred in most cases on the day of surgery (Table 4). Postsurgical exudation and edema in patients operated with excision-ablation technique was higher than in other groups. Most probably it is related to the time of exposure on laser light and consequently higher risk of thermal damage of surrounding tissues. Excessive peripheral thermal tissue effects may delay or create abnormal wound healing with increasing the possible risk of incisional dehiscence [4]. Wound healing delay is caused by temporary postponement of inflammation, phagocytic resorption, collagen production and re-epithelization in the early stages of repair [10]. Ablation was performed in cases when diameter of tumor did not exceed 0.5 cm. The time of exposure to the laser beam and consequently the risk of thermal injury was the lowest among the patients which explains the shortest time of healing in this group. Difference between extended time of healing between groups with excision and excision with ablation may also be caused by longer exposure to the laser beam.

**Table 4:** Prevalences of exudation and edema after tumor resection with different laser surgery technique

	**EXCISION**		**ABALTION**		**EXCISON/ABLATION**	
	**Edema [%]**	**Exudation [%]**	**Edema [%]**	**Exudation [%]**	**Edema [%]**	**Exudation [%]**
**Day 0**	21.4	0	20.0	0	43.8	0
**Day 3**	7.1	35.7	0	20.0	12.5	56.3
**Day 6**	0	28.6	0	0	0	12.5
**Day 12**	0	7.1	0	0	0	0

The basic principles of wide excision and removal of all abnormal tissue margins with CO_2_ laser should be the same as with conventional surgery technique [5,6]. During the 3-month post surgical observation period we noticed local recurrence only in two cases. Lanzafame et al. [11] demonstrated fewer recurrences after CO_2_ laser tumor removal compared to conventional scalpel excision. The probable cause of this difference is the sealing of small blood vessels and lymph vessels by the CO_2_ laser which prevents tumor cells spreading [3-5]. Moreover, both the no-touch surgery technique and the coagulation zone can reduce intraoperative wound contamination by malignant tumor cells what may result in decreasing recurrence risk.

Laser operation techniques by excision, ablation or excision with ablation provide better hemostasis, precision of working, non-contact dissection, less instruments at the site of operation and minimal traumatization of the surrounding tissues and an opportunity to reduce or eliminate risk of tumor recurrence. Good results of treatment depend on appropriate selection of technique adjusted to the particular case.

## Abbreviations

CO2: Carbon dioxide; CW: Continuous wave; SD: Spot diameter.

## Competing interests

The authors declare that they have no competing interests exist.

## Authors’ contributions

The study was designed by all authors. JP and JN did the surgery procedures and drafted the manuscript. ZK coordinated editing and revision of the manuscript. AA took pictures and anesthetized patients, MN and RC performed histopathological evaluation of the tumors and margins. All authors have read and approved the final version of the manuscript.
